# The draft genome sequence of the Brahminy blindsnake *Indotyphlops braminus*

**DOI:** 10.1038/s41597-022-01530-z

**Published:** 2022-07-15

**Authors:** Gulab Khedkar, Chiaki Kambayashi, Hiromasa Tabata, Ikuyo Takemura, Ryuhei Minei, Atsushi Ogura, Atsushi Kurabayashi

**Affiliations:** 1 Paul Hebert Centre for DNA Barcoding and Biodiversity Studies, Dr. Babasaheb Ambedkar Marathwada University, Aurangabad, Maharashtra India; 2 Department of Bio-Science, Nagahama Institute of Bio-Science and Technology, Shiga, Japan; 3 Unit for Environmental Sciences and Management, North-West University, Potchefstroom, South Africa

**Keywords:** Open reading frames, Next-generation sequencing

## Abstract

Blindsnakes of infraoder Scolecophidia (order Squamata) are the most basal group of extant snakes, comprising of more than 450 species with ecological and morphological features highly specialized to underground living. The Brahminy blindsnake, *Indotyphlops braminus*, is the only known obligate parthenogenetic species of snakes. Although the origin of *I. braminus* is thought to be South Asia, this snake has attracted worldwide attention as an alien species, as it has been introduced to all continents except Antarctica. In this study, we present the first draft genome assembly and annotation of *I. braminus*. We generated approximately 480 Gbp of sequencing data and produced a draft genome with a total length of 1.86 Gbp and N50 scaffold size of 1.25 Mbp containing 89.3% of orthologs conserved in Sauropsida. We also identified 0.98 Gbp (52.82%) of repetitive genome sequences and a total of 23,560 protein-coding genes. The first draft genome of *I. braminus* will facilitate further study of snake evolution as well as help to understand the emergence mechanism of parthenogenetic vertebrates.

## Abstract (summary)

**Table:** 

Measurement(s)	Whole genome sequence
Technology Type(s)	Next generation DNA sequencing
Factor Type(s)	Whole genome of Indotyphlops braminus
Sample Characteristic - Organism	Indotyphlops braminus
Sample Characteristic - Environment	Soil and rotten wood
Sample Characteristic - Location	Japan and India

## Background & Summary

The Infraorder Scolecophidia (blindsnakes) is the most basal lineage of extant snakes^1^. All constituent species are subterranean and are found mainly in the southern hemisphere and on tropical islands. They can range from 10 cm to nearly 1 m in length^2^, and they have highly specialized morphologies, including a vestigial organ form of eyes that can only perceive light. Although 462 species in five families have been described in Scolecophidia^3^, the true species diversity is thought to be greatly underestimated due to their cryptic ecology^4,5^.

As of April 2022, there are 32 available genome assemblies for snakes. Among the three major groups that comprise Serpentes (Caenophidia, Henophidia, and Scolecophidia), genomic data have been accumulated in Caenophidia, mainly for poisonous snakes belonging to the families Elapidae and Viperidae^6^ and in Henophidia, which includes the families Boidae and Pythonidae, for which the genome of *Python molurus bivittatus* has been reported^7^. However, there are currently no datasets for draft genome assemblies or annotations for snakes in the Scolecophidia group, despite the evolutionarily importance of this group, with the exception of low-quality assembly data (N50 < 2kbp)^8^.

**Fig. 1:**
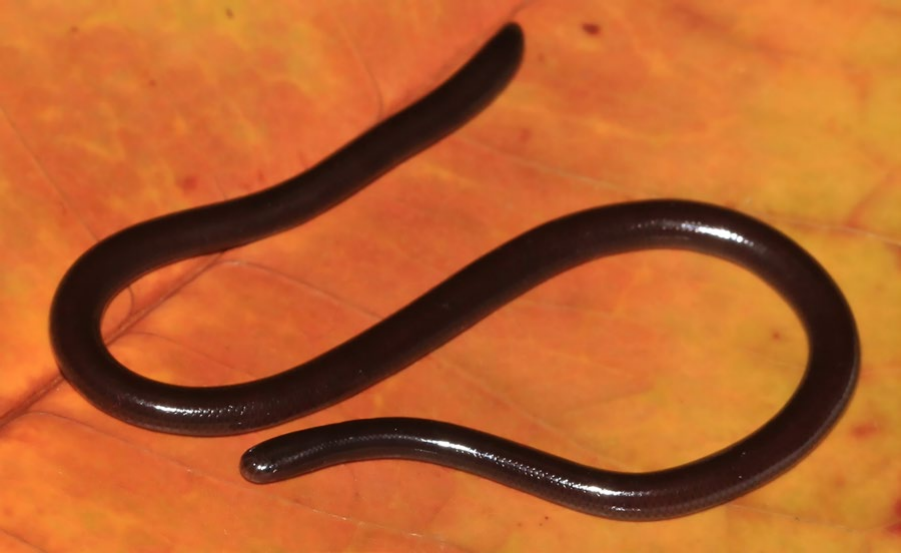
Live specimen of *Indotyphlops braminus*.

The Brahminy blindsnake, or *Indotyphlops braminus*, is one of the most well-known species in Scolecophidia (Fig. 1). No male *I. braminus* have been found, and this species of snake is the only known obligate parthenogenesis snake^9,10^. Further, *I. braminus* is an allotriploid (triploid) species^11–13^ and is considered to have emerged via inter-species hybridization, as has occurred with other parthenogenetic reptiles^14,15^. The geographic origin of this species is thought to be in South Asia based on the distribution of congeneric species^16,17^. However, due to their small size and fossorial and parthenogenetic nature, they have been transported around the world, hidden in the rotting woods and soils of ornamental plants. Consequently, *I. braminus* has now been colonized artificially and unintentionally in all continents except Antarctica^18,19^. Because *I. braminus* can be found globally, various studies regarding their osteology^20,21^, anatomy^22^, neurology^23^, and ethology^24,25^ have been conducted worldwide. For these reasons, *I. braminus* has the potential to serve as a useful snake model organism and is a suitable species in which to investigate the emergence mechanism of parthenogenesis in vertebrates.

In this study, we present the first draft genome of *I. braminus*. We extracted genomic DNA from liver and muscle tissues, constructed three pair-end (PE) libraries, and sequenced libraries using the Illumina Hiseq2500 platform. In addition, we conducted long-read sequencing of four libraries using Oxford Nanopore MinION and performed hybrid *de novo* assembly. The draft genome was assembled into 4,851 scaffolds (N50 = 1.25 Mbp) with a total size of 1.86 Gbp, comparable to the estimated genome size (1.50 Gbp) in k-mer analysis. Our BUSCO assessment indicated that 89.3% of orthologs conserved in Sauropsida were present in the genome assembly. Structural annotation of the genome identified 23,560 protein-coding genes. In the future, this highly-quality scolecophidian genome will be a crucial reference for further understanding of both snake evolution and the emergence mechanism of parthenogenetic species.

## Methods

### Sample Collection and DNA Extraction

We used two *I. braminus* specimens collected from India (Ooty: 11°24′26″ N, 76°41′27″ E) and Japan (Okinawajima Island: 26°15′09″N, 127°45′55″E), since *I. braminus* individuals are parthenogenetic clones, and the worldwide colonization of this blindsnake is thought to have occurred recently^26^. Indeed, the partial sequence of the mitochondrial cytochrome b gene of *I. braminus* from Japan (obtained by methods described previously in Smíd *et al*.^27^) matched perfectly with the corresponding region of the India specimen constructed by short-read data using NOVOPlasty v3.2^28^. The specimens used were picked up from under stones, euthanized, and dissected to isolate the liver and muscle tissues for DNA extraction. These experiments were performed under permissions received from the Ethics Committees for Animal Experiments by Dr. Babasaheb Ambedkar Marathwada University (permit No. A01) and Nagahama Institute of Bio-Science (permit No. 085).

For genome sequencing using Illumina, the *I. braminus* specimen from India was used, and DNA was extracted using the Wizard® Genomic DNA Purification Kit (Promega Corporation, WH, Madison, WI, USA). For Oxford Nanopore long-read sequencing, the specimen from Japan was used, and DNA extraction was performed using the Blood & Cell Culture DNA Midi Kit (Qiagen, Hilden, Germany) according to the manufacturer’s protocol. Purified precipitates were dissolved in TE buffer (pH 8.0) and stored at −30 °C until further processing.

### Library preparation and sequencing

Short-read sequencing libraries were prepared using the Illumina trueseq LT kit (Illumina, San Diego, CA, USA). Three PE libraries were prepared with an insert size of 550 bp and sequenced by Hiseq2500. Raw sequencing data were converted to fastq format using bcl2fastq2 v2.20. A total of 422 Gbp of sequences were obtained (Table 1), which were approximately 226.9 x coverage of *I. braminus* genome (1.86 Gbp, see below).

**Table 1:** Statistics of the sequencing data of *Indotyphlops braminus*.

platform	Average length (bp)	Raw bases (Gbp)	Raw reads	SRA accession
Illumina Hiseq	126	131.294	1,042,013,868	DRR374855^42^
	151	137.379	909,796,440	DRR374853^40^
	150.5	153.493	1,020,049,008	DRR374854^41^
Total	—	422.166	2,971,859,316	—
Oxford Nanopore MinION	4,810.6	14.518	3,017,890	DRR374856^43^
	6,524.3	18.457	2,829,032	DRR374857^44^
	6,218.9	15.241	2,450,721	DRR374858^45^
	7,048.1	9.732	1,380,757	DRR374859^46^
Total	—	57.948	9,678,400	—

For long-read sequencing using MinION (Oxford Nanopore Technology, Oxford, UK), the extracted genomic DNA was fragmented to ~20 kbp using Covaris g-TUBE (Covaris, Woburn, MA, USA). After purification using 0.4 x AMPure XP beads (Beckman Coulter, Brea, CA, USA), library preparation was performed using the SQK-LSK109 Ligation Sequencing kit (Oxford Nanopore Technologies) based on the manufacturer’s protocol. Four libraries were prepared and loaded onto R9.4.1 chemistry flowcell (FLO-MIN106) and sequenced using MinKNOW v 19.06.7. After sequencing, Guppy v3.2.2 was used for basecalling. A total of 57.9 Gbp of long-read data were obtained (Table 1), which were 31.1 x coverage of *I. braminus* genome. The raw reads were checked using LongQC v1.2.0c^29^, and quality filtered using Filtlong v0.2.1 (https://github.com/rrwick/Filtlong) with a minimum QV of 10 and a minimum read length of 1 Kbp.

### Genome assembly

**Fig. 2:**
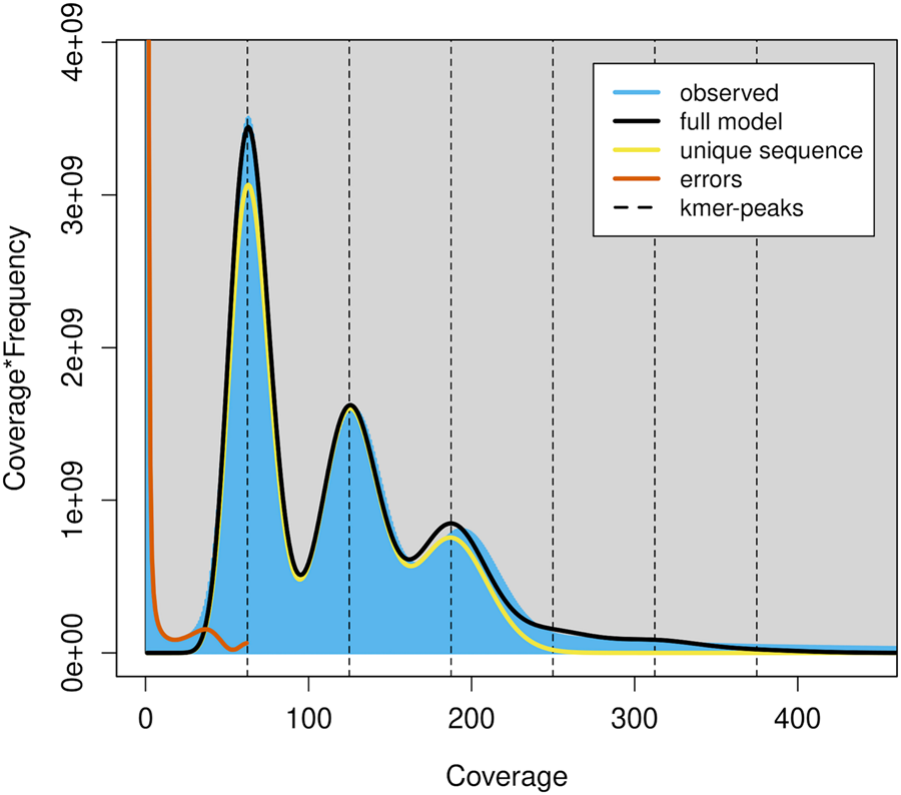
The k-mer distribution (k = 21) of *Indotyphlops braminus*. The 21-mer distribution was calculated by GenomeScope based on 422 Gbp Illumina short-reads data. K-mer coverages (x axis) were plotted against the value of coverage multiplying frequency (y axis).

We estimated the overall characteristics of the *I. braminus* genome, including its genome size, heterozygosity, and repeat content, by k-mer frequencies calculated from Illumina short-reads. KMC v3.1.1^30^ was used to obtain a 21-mer count histogram (Fig. 2). GenomeScope v2.0^31^ estimated a genome size of 1.50 Gbp, which was comparable with that of our draft genome (1.86 Gbp). The genome size of *I. braminus* fell within the range of other snake species whose genomes have been reported previously (1.13–2.03 Gbp).

We applied a hybrid *de novo* assembly approach based on Illumina short-reads and Nanopore long-reads. Short- and long-reads were assembled to contigs using MaSuRCA v4.0.5^32^. For gap-closing, assembled contigs were scaffolded into the draft genome using HaploMerger2 v20180603^33^. The resultant draft genome had a total length of 1.86 Gbp, scaffold number of 4,851, N50 of 1.25 Mbp and the longest scaffold of length 7.0 Mbp, as calculated by QUAST v5.0.2^34^ (Table 2). We evaluated the gene completeness of our draft genome using BUSCO v5.2.2^35,36^. BUSCO assessment showed that 89.3% of orthologs conserved in Sauropsida were present in this genome assembly (sum of the percentages of single-copy and duplicate), suggesting that our draft genome possessed a sufficient gene repertoire from *I. braminus* (Table 2).

**Table 2:** Statistics of the genome assembly.

Scaffolds	4,851
Maximum length (bp)	7,047,253
Total length (bp)	1,856,433,866
N50 (bp)	1,247,154
GC%	41.96
BUSCO complete (%)	89.3
BUSCO single-copy (%)	87.4
BUSCO duplicated (%)	1.9

### Repeat analysis

Repetitive regions of *I. braminus* were identified using a combination of *de novo* and homology-based approaches. For homology-based prediction, known repetitive elements were identified using RepeatMasker v4.1.1 (http://www.repeatmasker.org) to search against published RepBase sequences. For *de novo* prediction, RepeatModeler v2.0.1 was executed on the *I. braminus* assembly to build a *de novo* repeat library for this species. Then, RepeatMasker was used to annotate repetitive elements using this library. The estimated repeat regions of total length 0.98 Gbp accounted for 52.82% of the genome. Long interspersed nuclear elements were the most abundant elements and accounted for 20% of the genome. A summary of the annotation is shown in Table 3.

**Table 3:** Statistics of repeat elements in the genome of *Indotyphlops braminus*.

Repeat elements	Copies	Length (bp)	Percent (%)
SINE	114704	14502484	0.78
LINE	1120299	371407334	20.01
LTR elements	81181	80025476	4.31
DNA elements	165204	33202527	1.79
Unclassified	2257178	461138007	24.84
Small RNA	10022	791165	0.04
Satellites	245	33662	0
Simple repeats	314143	15855113	0.85
Low complexity	45366	3575448	0.19
Total	4108342	980531216	52.82

### Gene prediction and annotation

**Fig. 3:**
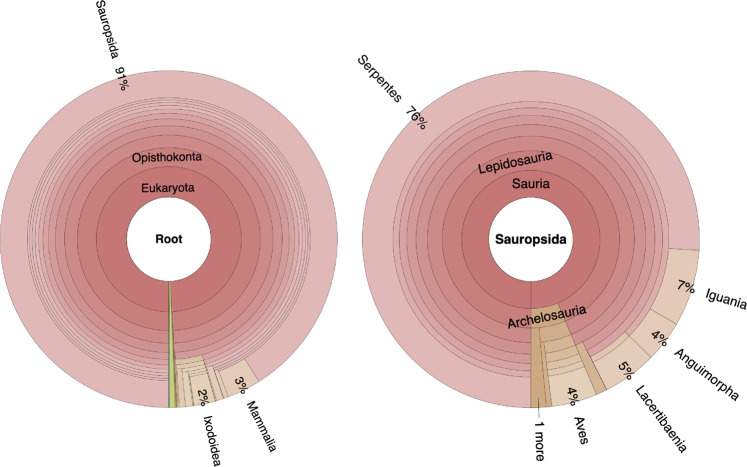
Krona chart representing taxonomic composition of *Indotyphlops braminus* gene model. Taxonomy charts, which consist of all taxa (left) and Sauropsida (right), are shown.

A BLAST search with the known mitochondrial DNA sequence of *I. braminus* (Accession number: NC_010196) identified a contig showing 99.9% homology. This was a mitochondrial DNA excluded from the assembly data. We also masked repeat regions and conducted gene prediction using Augustus v3.4.0^37^ trained with the assessment result of BUSCO with respect to the genome assembly. In total, 23,560 protein-coding genes were annotated in the *I. braminus* genome (Table 4). Next, we investigated the closest protein homolog of each entry in the gene model of *I. braminus* using diamond v2.0.13^38^, and visualized results by Krona^39^ (Fig. 3). Approximately 91% of the closest protein homolog of each gene of the gene model belonged to Sauropsida. Of the proteins detected in Sauropsida, approximately 76% were derived from Serpentes, indicating that the gene model is quite consistent with the systematic position of *I. braminus*.

**Table 4:** Statistics of the gene model of *Indotyphlops braminus*.

Number of protein-coding genes	23,560
Average CDS length (bp)	20,067.5
Average exon number per gene	7.7
Average exon length (bp)	188.2
Average intron length (bp)	2,788.1
BUSCO complete (%)	72.9
BUSCO single-copy (%)	71.4
BUSCO duplicated (%)	1.5

The BUSCO analysis with Sauropsida conserved genes databases found 72.9% completeness in our annotation dataset (Table 4), which was lower than that estimated in the genome assembly (89.3%: Table 2). Since the completeness of predicted genes was evaluated based on the codon reading frame, it is likely that there were low-quality genes exhibiting premature termination. In this analysis, we applied a hybrid assembly with short-reads (accuracy >99.9%) and long-reads (<85%), which may have resulted in a lower base accuracy for the assembled regions with only long-reads and in low BUSCO value. To improve the assembly of the *I. braminus* genome, it would be necessary to obtain novel transcriptome data or perform further high accuracy short- and long-read sequencing.

## Data Records

All DNA raw reads have been deposited in the NCBI SRA^40–46^ (Table 1) with the accession code (Bioproject) PRJDB13523.

## Technical Validation

### Quality assessment of the genome assembly

The total assembly length is 1.86 Gbp, which is almost comparable with the estimated genome size (1.50 Gbp). The scaffold N50 is 1.25 Mbp (Table 2). BUSCO analysis was performed with Sauropsida conserved genes databases to assess the completeness of the genome assembly, resulting in a BUSCO value of 89.3%.

### Gene prediction and annotation validation

Gene models in the assembly were predicted using Augustus trained with the BUSCO assessment result. The final gene set consisted of 23,560 genes (Table 4). The BUSCO value was 72.9%, which was lower than that in the genome assembly, probably due to the insufficient reliability of the regions assembled using only long-reads data.

## Data Availability

All analyses were conducted on Linux systems. The version and code and parameters of the main software tools are described below. (1) LongQC, version 1.2.0c, parameters used: default. (2) Filtlong, version 0.2.1, parameters used: min_length 1000, keep_percent 90, split 100, mean_q_weight 10. (3) KMC, version 3.1.1, parameters used: k21, ci1, cs10000. (4) GenomeScope, version 2.0, parameters used: ploidy 3, kmer_length 21. (5) MaSuRCA, version 4.0.5, parameters used: LIMIT_JUMP_COVERAGE = 300, CA_PARAMETERS = cgwErrorRate = 0.15, FLYE_ASSEMBLY = 0. (6) HaploMerger2, version 20180603, parameters used: default; hm.batchA and hm.batchB. (7) QUAST, version 5.0.2, parameters used: default. (8) BUSCO, version 5.2.2, parameters used: lineage_dataset sauropsida_odb10. (9) RepeatMasker, version 4.1.1, parameters used: engine ncbi, xsmall, Database: Dfam with RBRM. (10) RepeatModeler, version 2.0.1, parameters used: default, Database: The scaffolds assembled with MaSuRCA and HaploMerger2. (11) Augustus, version 3.4.0, parameters used: species = Database trained with BUSCO, alternatives-from-evidence = true, hintsfile = Output of RepeatMasker. (12) Diamond, version 2.0.13, parameters used: more-sensitive, max-target-seqs. 1, evalue 1e-5.
